# From patterns to prognosis: machine learning–derived clusters in advanced heart failure

**DOI:** 10.3389/fcvm.2025.1669538

**Published:** 2025-10-23

**Authors:** Murat Karaçam, Barkın Kültürsay, Deniz Mutlu, Seda Tanyeri, Azmican Kaya, Süleyman Çagan Efe, Cem Doğan, Gülümser Sevgin Halil, Özgür Yaşar Akbal, Kaan Kırali, Rezzan Deniz Acar

**Affiliations:** ^1^ Department of Cardiology, Bitlis State Hospital, Bitlis, Türkiye; ^2^ Department of Cardiology, Tunceli State Hospital, Tunceli, Türkiye; ^3^ Center for Coronary Artery Disease, Minneapolis Heart Institute Foundation, Minneapolis, MN, United States; ^4^ Department of Cardiology, Kartal Kosuyolu Research and Education Hospital, Istanbul, Türkiye; ^5^ Department of Cardiovascular Surgery, Kartal Kosuyolu Research and Education Hospital, İstanbul, Türkiye

**Keywords:** advanced heart failure, phenotyping, unsupervised clustering, machine learning, risk stratification

## Abstract

**Introduction:**

Advanced heart failure (HF) is a clinically heterogeneous condition with poor prognosis, and traditional classification systems often fail to capture the complexity needed for personalized care. This study aimed to identify clinically meaningful phenotypic subgroups among patients with advanced HF using unsupervised machine learning and to evaluate their association with long-term outcomes.

**Methods:**

A retrospective analysis was conducted on 524 patients with advanced HF who underwent comprehensive clinical, echocardiographic, hemodynamic, and cardiopulmonary exercise assessments. Using k-means clustering on standardized, multidimensional data, two distinct phenotypes were identified. The primary composite outcome was defined as all-cause mortality, left ventricular assist device implantation, or heart transplantation. Associations between cluster assignment and outcomes were evaluated using Kaplan–Meier analysis and Cox proportional hazards regression.

**Results:**

The first cluster, representing patients with relatively preserved hemodynamics and functional status, was associated with a more favorable prognosis, while the second cluster included older individuals with significant biventricular dysfunction, higher pulmonary pressures, and poorer exercise capacity. These patients experienced a markedly higher rate of the composite outcome over a median follow-up of 2.4 years, with Cluster 2 showing a significantly increased risk (hazard ratio [HR]: 3.84; 95% CI: 2.72–5.43; *p* < 0.001).

**Conclusion:**

Machine learning–based clustering revealed two distinct phenotypes in advanced HF with differing clinical features and prognoses. This approach may enhance risk stratification and inform individualized therapeutic strategies in this high-risk population.

## Introduction

Heart failure (HF) is a complex clinical syndrome characterized by substantial heterogeneity in etiology, pathophysiology, disease trajectory, and response to therapy. This heterogeneity becomes particularly evident in patients with advanced HF, a population that remains underrepresented in large-scale clinical trials despite experiencing the highest rates of morbidity and mortality (1). The prevalence of this patient group continues to rise due to both an aging global population and the increasing availability of life-prolonging therapies (2). These patients also represent a significant burden on healthcare systems, largely due to frequent hospital readmissions and progressive clinical deterioration (3).

Traditional classifications of heart failure—based on subjective measures of functional status, left ventricular ejection fraction (LVEF) thresholds, or broad stage designations (A to D)—are insufficient to reflect the phenotypic complexity observed in clinical practice (2–4). Recent advances in machine learning (ML) have enabled novel phenotyping strategies, shifting from reductionist models to multidimensional frameworks that incorporate clinical, imaging, and biomarker data (5, 6). In particular, unsupervised learning methods have facilitated the identification of latent subgroups—so-called “phenoclusters”—within heterogeneous HF populations. These data-driven approaches do not rely on pre-labeled outcomes, allowing for the unbiased discovery of previously unrecognized clinical patterns and their prognostic implications (7, 8).

The clinical relevance of phenotypic clustering is increasingly recognized, as subgroups show differing treatment responses and outcomes (9). In heart failure with preserved ejection fraction (HFpEF)—the most extensively studied patient population—phenomapping has identified reproducible clusters linked to comorbidities, structural remodeling, and exercise intolerance (6, 10, 11). However, advanced HF, despite its distinct pathophysiology and poor prognosis, remains underrepresented in such studies (12). The complexity of therapy selection, including transplantation and left ventricular assist device (LVAD), underscores the need for robust stratification models, yet ML applications in this population are still limited.

This study had two main objectives: to identify phenotypic clusters among patients with advanced HF using unsupervised ML techniques, and to assess the prognostic significance of these clusters.

## Materials and methods

### Study population

A total of 653 consecutive patients with advanced heart failure, defined according to the 2021 European Society of Cardiology (ESC) Guidelines as having persistent severe symptoms (NYHA class III–IV) with objective evidence of cardiac dysfunction and poor prognosis despite optimal medical therapy, and who were referred to our tertiary cardiovascular center for evaluation of advanced therapeutic options (including LVAD and transplantation), were initially evaluated between January 2021 and April 2024 (2). Patients with prior durable LVAD implantation, previous heart transplantation, left ventricular ejection fraction (LVEF) > 25%, severe pulmonary disease, contraindications to CPET or RHC, or incomplete follow-up data were excluded. After applying these exclusion criteria, 524 patients constituted the final study cohort (Supplementary Figure S1). All included patients underwent comprehensive baseline evaluation with transthoracic echocardiography, cardiopulmonary exercise testing (CPET), and right heart catheterization (RHC), performed within a 14-day time window. All demographic, clinical, laboratory, echocardiographic, and hemodynamic variables were obtained from the hospital's electronic medical record (EMR) system. Clinical diagnoses were determined based on International Classification of Diseases (ICD) codes and subsequently verified through physician notes and laboratory reports to ensure accuracy. CPET parameters were extracted through additional manual chart review of exercise test reports by the investigator team. Standardized definitions were applied in line with established guidelines: diabetes mellitus (DM) was defined as a physician-documented diagnosis and/or use of antidiabetic medication (13); atrial fibrillation (AF) as documented arrhythmia on ECG or Holter monitoring (14); ischemic etiology as a history of myocardial infarction, percutaneous coronary intervention, or coronary artery bypass grafting; hypertension (HT) as a physician-documented diagnosis and/or use of antihypertensive therapy (15); hyperlipidemia (HL) as a physician-documented diagnosis and/or use of lipid-lowering therapy; chronic kidney disease (CKD) as an estimated glomerular filtration rate <60 ml/min/1.73 m^2^ persisting for >3 months (16); cerebrovascular disease (CVD) as a history of ischemic or hemorrhagic stroke or transient ischemic attack; and chronic obstructive pulmonary disease (COPD) as a physician-documented chronic airway disease with or without pulmonary function testing.

The study was approved by the local ethics committee and conducted in accordance with the Declaration of Helsinki.

### Echocardiography

LVEF was measured using the biplane method of disks summation (modified Simpson's rule). Doppler echocardiographic examinations were performed by a single experienced cardiologist using the EPIQ CVx version 9.0.5 system and both S5-1 and X5-1 transducers (Philips Medical Systems, Andover, MA, USA), in accordance with current guidelines. Tricuspid annular plane systolic excursion (TAPSE) was obtained using M-mode imaging from the apical four-chamber view with focus on the right ventricle. Pulmonary artery systolic pressure (PASP) was estimated by adding the peak tricuspid regurgitant jet velocity (using the Bernoulli equation) to the estimated central venous pressure, which was derived from the diameter and respiratory variation of the inferior vena cava (IVC). All echocardiographic measurements adhered to the recommendations of the American Society of Echocardiography (17).

### Exercise testing

Maximal cardiopulmonary exercise testing was performed using a continuous, individualized ramp treadmill protocol on a JAEGER Vyntus CPX system (Vyaire Medical, Germany). Exercise capacity was expressed in metabolic equivalents (METs), with oxygen uptake (VO_2_) measured breath by breath through an automated system. Measurements were recorded at rest, throughout graded exercise, and during a two-minute recovery period. METs were calculated by dividing VO_2_max by 3.5 ml/kg/min. VO_2_, VCO_2_, and the respiratory exchange ratio (RER = VCO_2_/VO_2_) were averaged every 10 s. Peak VO_2_ was defined as the highest 10 s averaged VO_2_ during the final stage of exercise. Blood pressure was measured prior to testing and at three-minute intervals throughout the protocol and recovery.

### Cardiac catheterization

Right heart catheterization was performed via the right internal jugular or femoral vein using a 7Fr balloon-tipped Swan–Ganz catheter (Edwards Lifesciences, Irvine, CA, USA) or a pigtail catheter. Cardiac output was calculated using the indirect Fick method. All pressure waveforms were visually assessed to ensure physiological accuracy, and measurements were taken at end-expiration.

### Endpoint definition

The composite outcome was defined as all-cause mortality, LVAD implantation, or heart transplantation, in line with definitions used in previous literature (18, 19).

### Statistical analysis

**Figure 1:**
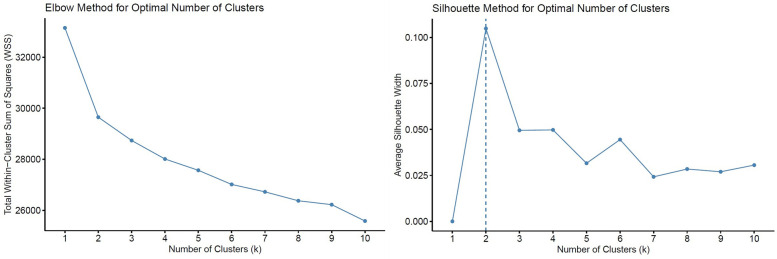
Determination of the optimal number of clusters (*k*) using the elbow and silhouette methods. Total within-cluster sum of squares (WSS) plotted against increasing values of k. The elbow point was visually identified at *k* = 2, where the reduction in WSS began to plateau. Average silhouette width across varying k values, with the highest value observed at *k* = 2, supporting the two-cluster solution.

**Figure 2:**
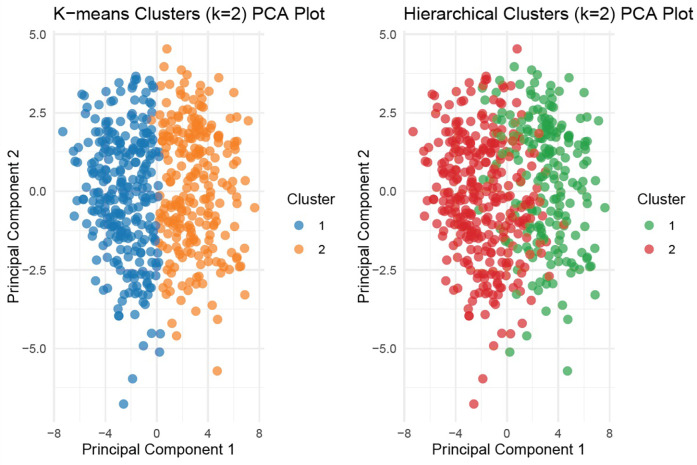
Principal component analysis (PCA) for *k*-means and hierarchical clustering (*k* = 2). Visualization of patient distribution by unsupervised clustering (*k*-means and hierarchical) using first two principal components. Distinct separation is evident between the two clusters in *k*-means clustering.

**Figure 3:**
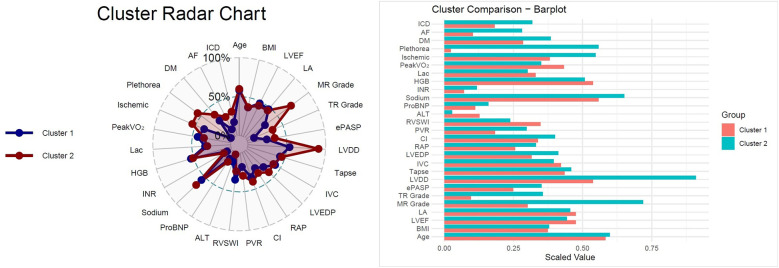
Cluster radar chart and bar plot comparison*,*radar chart illustrating normalized distributions of selected parameters across the two clusters. Cluster 2 demonstrated greater impairments in hemodynamic, biochemical, and echocardiographic variables compared with Cluster 1. Bar plot comparing scaled mean values for clinical, echocardiographic, laboratory, and hemodynamic parameters between clusters. Cluster 2 was characterized by older age, higher prevalence of comorbidities (diabetes mellitus, atrial fibrillation), worse hemodynamics (higher RAP, LVEDP, and PVR; lower CI), and impaired functional status (lower peak VO_2_).

To identify distinct phenotypic clusters within the study population, we employed unsupervised machine learning techniques. Prior to clustering, missing data were addressed via the *MissForest* algorithm, a non-parametric, iterative imputation method utilizing random forests (20) (Supplementary Figure S2). All continuous variables were standardized to zero mean and unit variance prior to distance-based modeling. Binary categorical variables (e.g., comorbidities, sex) were excluded from the clustering process to prevent distortion in Euclidean distance calculations arising from incompatible data types. Ordinal categorical variables (e.g., mitral regurgitation grade, tricuspid regurgitation grade, and LV diastolic dysfunction) were converted to integer scores respecting their inherent order, thereby preserving their rank information in the distance matrix. A total of 108 variables were considered, encompassing clinical, laboratory, echocardiographic, hemodynamic, and CPET parameters. After addressing multicollinearity (removing one variable from each pair with Pearson correlation >0.7 based on clinical judgment), 81 variables remained for the final clustering analysis (Supplementary Table S1). Both hierarchical clustering (Ward's method with Euclidean distance) and k-means clustering were applied to the scaled numeric data. These algorithms are well suited for standardized continuous data and have been widely applied in heart failure phenomapping studies (5, 6). The optimal number of clusters was determined using both the elbow method (within-cluster sum of squares) and the average silhouette width as complementary approaches (Figure 1). The elbow point was visually identified at *k* = 2, where the incremental reduction in WSS plateaued, and this was further supported by the highest silhouette score. While *k* = 3 showed a minor secondary inflection, it yielded a lower silhouette width and produced less stable, clinically interpretable clusters. Hierarchical clustering provided an interpretable dendrogram and stable grouping (Supplementary Figure S3); however, *k*-means clustering demonstrated comparable or higher silhouette scores, offering more flexible partitioning and iterative refinement (Figures 1, 2). Therefore, *k*-means clustering (*k* = 2) was selected for the final classification (Supplementary Figures S4, S5), balancing statistical performance, model simplicity, and clinical interpretability. To evaluate the robustness of the identified clusters, internal validation was performed using bootstrap resampling with 1,000 iterations and Jaccard similarity indices. As a sensitivity analysis, clustering was repeated using Gower distance with partitioning around medoids (PAM) (Supplementary Figure S6). Additionally, internal validation was performed using the Calinski–Harabasz (CH) and Davies–Bouldin (DB) indices across different cluster numbers (*k* = 2–6) (Supplementary Table S2). The final cluster assignments were appended to the imputed dataset. Group differences between clusters were assessed using chi-squared tests for categorical variables and either Student's *t*-test or Wilcoxon rank-sum test for continuous variables, depending on distributional assumptions. Scaled variables were compared between the two clusters using both bar plots and a radar chart to illustrate group-level differences (Figure 3). Survival was illustrated using the Kaplan–Meier method, and Cox proportional hazards regression models were applied to assess time-to-event associations between cluster membership and outcomes. Importantly, outcomes were not included as clustering inputs, ensuring independence between phenotype derivation and prognostic evaluation. The proportional hazards assumption was tested using Schoenfeld residuals and was not violated (Supplementary Figure S7). To further assess the reproducibility of the clustering solution, repeated split-sample validation was performed. In each of 100 random replications, the cohort was divided into 70% training and 30% validation subsets. K-means clustering (*k* = 2) was derived in the training set, and cluster centroids were used to assign patients in the validation set. Agreement between original and validation cluster assignments was quantified by the adjusted Rand index, while prognostic validity was evaluated using log-rank tests and Cox regression (Supplementary Table S3). All statistical tests were two-tailed, and a *p*-value below 0.05 was considered statistically significant. All statistical analyses were performed using the R 4.4.1 software (R Foundation for Statistical Computing, Vienna, Austria) with packages “missForest”, “dplyr”, “stats”, “cluster”, “clusterCrit”, “fossil”, “naniar”, “dendextend”, “survival”, “survminer”, “rms”, “ggplot2”.

## Results

### Cluster validation

Unsupervised *k*-means clustering identified two distinct phenotypic clusters among 524 patients with advanced HF. For the primary *k*-means model based on continuous variables, both clusters showed excellent stability (Jaccard indices: 0.998 and 0.985; Supplementary Figure S6). Sensitivity analysis using Gower distance with PAM clustering yielded consistent results, although with moderately lower stability (Jaccard indices: 0.851 and 0.762). Internal validation using the Calinski–Harabasz (CH) and Davies–Bouldin (DB) indices across different cluster numbers (*k* = 2–6) consistently supported the two-cluster solution, which yielded the highest CH and the lowest DB values (Supplementary Table S2).

Split-sample validation further confirmed reproducibility. Across 100 replications of 70/30 splits, the adjusted Rand index averaged 0.77 ± 0.06, and prognostic separation was consistently observed (log-rank *p* < 0.05 in all subsets; pooled HR: 0.83, 95% CI: 0.78–0.89; Supplementary Table S3). Notably, this validation HR reflects reproducibility across resampling iterations, whereas the full-cohort Cox model showed the absolute effect size (HR: 3.84, 95% CI: 2.72–5.43).

Collectively, these analyses indicate that the identified phenotypes are reproducible, robust, and prognostically meaningful rather than artifacts of overfitting.

Cluster 1 comprised 282 patients (53.8%), while Cluster 2 included 242 patients (46.2%). Based on their clinical and physiological profiles, we defined Cluster 1 as the **Favorable Profile Cluster (FPC)**, characterized by more favorable hemodynamic and functional parameters—suggestive of a group appropriate for continued monitoring and optimization of standard therapies. In contrast, Cluster 2 was designated the **Adverse Profile Cluster (APC)**, representing an older cohort with marked hemodynamic compromise and diminished exercise capacity, indicative of a phenotype that may benefit from earlier consideration of advanced interventions or intensified medical management.

### Clinical and demographic characteristics

Patients in Cluster 2 were older (median age: 54 (45–60) vs. 52 (43–58) years, *p* = 0.013) and had a lower body mass index (BMI: 27.0 ± 4.8 vs. 28.2 ± 5.3 kg/m^2^, *p* = 0.005) (Table 1). There was no significant difference in sex distribution between the two clusters. The prevalence of ischemic etiology (54.7% vs. 38.2%, *p* < 0.001), history of percutaneous coronary intervention (45.9% vs. 28.5%, *p* < 0.001), coronary artery bypass grafting (14.5% vs. 7.8%, *p* = 0.015), diabetes mellitus (38.4% vs. 28.5%, *p* = 0.016), atrial fibrillation (28.1% vs. 10.3%, *p* < 0.001), and implantable cardioverter defibrillator (31.8% vs. 18.1%, *p* < 0.001) was significantly higher in Cluster 2.

**Table 1:** Demographic data of patients.

Variable	Overall (*n* = 524)	Cluster 1 (*n* = 282)	Cluster 2 (*n* = 242)	*p*
Gender (male)	447 (85.3)	241 (85.5)	206 (85.1)	0.913
Age (years)	53 (44–59)	52 (43–58)	54 (45–60)	0.013
BMI (kg/m^2^)	27.6 (5.1)	28.2 (5.3)	27 (4.8)	0.005
Ischemic etiology	231 (44.1)	102 (38.2)	129 (54.7)	<.001
PCI	191 (36.5)	80 (28.5)	111 (45.9)	<.001
CABG	57 (10.9)	22 (7.8)	35 (14.5)	0.015
HT	185 (35.3)	102 (36.3)	83 (34.3)	0.633
DM	173 (33.0)	80 (28.5)	93 (38.4)	0.016
AF	97 (18.5)	29 (10.3)	68 (28.1)	<.001
HL	209 (39.9)	107 (38.1)	102 (42.1)	0.343
CKD	102 (19.5)	49 (17.4)	53 (21.9)	0.199
CVD	42 (8.0)	21 (7.5)	21 (8.7)	0.613
PAD	26 (5.0)	10 (3.6)	16 (6.6)	0.109
Smoker	371 (70.8)	196 (69.8)	175 (72.3)	0.520
COPD	60 (11.5)	30 (10.7)	30 (12.4)	0.538
ICD	128 (24.4)	51 (18.1)	77 (31.8)	<.001
CRT	33 (6.3)	19 (6.8)	14 (5.8)	0.647

AF, Atrial Fibrillation; BMI, Body Mass Index; CABG, Coronary Artery Bypass Graft; CKD, Chronic Kidney Disease; COPD, Chronic Obstructive Pulmonary Disease; CRT, Cardiac Resynchronization Therapy; CVD, Cerebrovascular disease; DM, Diabetes mellitus; HL, Hyperlipidemia; HT, Hypertension; ICD, Implantable Cardioverter Defibrillator; LVAD, Left ventricular assist device; PAD, Peripheral artery disease; PCI, Percutaneous Coronary Intervention; Tx, Heart transplantation.

### Echocardiographic and hemodynamic findings

Table 2 demonstrates the echocardiographic findings of the patients. Cluster 2 demonstrated more advanced structural and functional cardiac abnormalities. Left ventricular ejection fraction (LVEF) was significantly lower in Cluster 2 (median: 20% (18–24) vs. 23% (20–25), *p* < 0.001), suggesting more profound systolic dysfunction. Although left ventricular end-diastolic and end-systolic diameters (LVEDD, LVESD) were similar between groups, left atrial size was markedly increased in Cluster 2 (4.84 ± 0.53 cm vs. 4.45 ± 0.61 cm, *p* < 0.001), reflecting chronic volume overload and diastolic impairment.

**Table 2:** Echocardiographic parameters.

Variable	Overall (*n* = 524)	Cluster 1 (*n* = 282)	Cluster 2 (*n* = 242)	*p*
LVEF (%)	22 (20–25)	23 (20–25)	20 (18–24)	<.001
LVEDD (cm)	6.7 (6.2–7.4)	6.7 (6.2–7.35)	6.8 (6.2–7.4)	0.621
LVESD (cm)	5.9 (5.4–6.6)	5.8 (5.3–6.57)	6 (5.5–6.6)	0.106
LA (cm)	4.63 (0.61)	4.45 (0.61)	4.84 (0.53)	<.001
MR				
Grade 1	183 (34.9)	143 (52.2)	40 (17.0)	<.001
Grade 2	210 (40.1)	97 (35.4)	113 (49.1)	
Grade 3	111 (21.2)	34 (12.4)	77 (33.5)	
TR				
Grade 1	286 (54.6)	229 (82.7)	57 (23.8)	<.001
Grade 2	154 (29.4)	43 (15.5)	111 (46.3)	
Grade 3	77 (14.7)	5 (1.8)	72 (29.7)	
Echo PASP (mmHg)	40 (30–53)	30 (25–40)	50 (42–63)	<.001
LVDD				
Grade 1	101 (19.3)	85 (31.7)	16 (6.6)	<.001
Grade 2	106 (20.2)	78 (29.1)	28 (12.4)	
Grade 3	287 (54.8)	105 (39.2)	182 (80.5)	
TAPSE (cm)	1.68 (0.46)	1.8 (0.44)	1.4 (0.37)	<.001
IVC (cm)	1.91 (0.49)	1.64 (0.35)	2.23 (0.43)	<.001
Plethora	132 (25.2)	6 (2.3)	126 (55.8)	<.001

LA, Left atrium; LVDD, Left ventricular diastolic dysfunction; LVEDD, Left ventricular end-diastolic diameter; LVEF, Left ventricular ejection fraction; LVESD, Left ventricular end-systolic diameter; MR, Mitral regurgitation; PASP, Pulmonary artery systolic pressure; TAPSE, Tricuspid annular plane systolic excursion; TR, Tricuspid regurgitation.

Mitral regurgitation severity was significantly greater in Cluster 2, with higher proportions of patients exhibiting moderate-to-severe regurgitation (Grade 2–3 in 82.6% vs. 47.8%, *p* < 0.001). Similarly, tricuspid regurgitation was more severe in Cluster 2, indicating substantial right-sided valvular involvement and volume burden.

Right ventricular systolic function was also significantly impaired in Cluster 2, with lower tricuspid annular plane systolic excursion (TAPSE: 1.4 ± 0.37 cm vs. 1.8 ± 0.44 cm, *p* < 0.001) and increased inferior vena cava (IVC) diameter (2.23 ± 0.43 cm vs. 1.64 ± 0.35 cm, *p* < 0.001), suggesting elevated right atrial pressures and reduced RV contractility. Plethora was observed in over half of Cluster 2 patients (55.8% vs. 2.3%, *p* < 0.001). Estimated pulmonary artery systolic pressure (PASP) by echocardiography was higher in Cluster 2 (median: 50 mmHg vs. 30 mmHg, *p* < 0.001), consistent with pulmonary hypertension.

Invasive hemodynamic assessment via right heart catheterization revealed marked elevation in biventricular filling pressures and pulmonary vascular resistance in Cluster 2 (Table 3). Left ventricular end-diastolic pressure (LVEDP), along with pulmonary artery systolic, diastolic, and mean pressures were significantly higher in Cluster 2 than Cluster 1. Cluster 2 also exhibited elevated right atrial pressure (RAP: 12 (9–17) vs. 6 (4–8) mmHg, *p* < 0.001), right ventricular systolic pressure (RVSP: 59 (48–71) vs. 36 (29–49) mmHg, *p* < 0.001), and transpulmonary gradient (TPG: 12 (8–19) vs. 6 (3–9) mmHg, *p* < 0.001) than Cluster 1, indicating more frequent combined pre- and post-capillary pulmonary hypertension in Cluster 2.

**Table 3:** Cardiac catheterization parameters.

Variable	Overall& (*n* = 524)	Cluster 1 (*n* = 282)	Cluster 2 (*n* = 242)	*p*
Aortic Systolic Pressure (mmHg)	117.3 (25.3)	123.2 (26.6)	111.2 (22.3)	<.001
Aortic Diastolic Pressure (mmHg)	71.4 (13.8)	72.1 (14.5)	70.7 (12.9)	0.314
Aortic Mean Pressure (mmHg)	87.5 (15.6)	89.9 (15.9)	85.1 (14.9)	0.002
LVEDP (mmHg)	24 (15–28)	17 (12–24)	27 (23–30)	<.001
Cath PASP (mmHg)	50 (35–63)	36 (28–50)	60 (50–72)	<.001
Cath PADP (mmHg)	23 (14–29)	14 (10–21)	28 (24–33)	<.001
Cath PAMP (mmHg)	33 (22–42)	23 (17–32)	40 (36–46)	<.001
Cath RVSP (mmHg)	48 (36–62)	36 (29–49)	59 (48–71)	<.001
Cath RAP (mmHg)	8 (5–14)	6 (4–8)	12 (9–17)	<.001
TPG (mmHg)	9 (5–14)	6 (3–9)	12 (8–19)	<.001
TSG (mmHg)	77.8 (16.3)	83.3 (15.5)	72.2 (15.1)	<.001
Stroke Volume (ml/beat)	40.02 (32.31–51.00)	47.00 (39.00–57.59)	35.00 (29.20–41.00)	<.001
Stroke Volume Index (ml/m^2^/beat)	20.80 (17.12–25.66)	23.80 (19.98–28.32)	18.00 (15.20–21.22)	<.001
Aortic Oxygen Saturation (%)	96.1 (2.4)	96.5 (2.1)	95.7 (2.6)	<.001
MPA Oxygen Saturation (%)	55.6 (10.7)	63.0 (7.0)	48.2 (8.3)	<.001
Cardiac Output (L/min)	3.32 (2.80–4.12)	3.94 (3.22–4.54)	3.00 (2.50–3.45)	<.001
Cardiac Index (L/min/m^2^)	1.70 (1.50–2.06)	1.92 (1.66–2.25)	1.57 (1.32–1.78)	<.001
PVR (Woods Unit)	2.45 (1.36–4.30)	1.40 (0.90–2.33)	4.10 (2.56–6.40)	<.001
SVR (Woods Unit)	22.80 (18.96–27.00)	21.40 (17.60–25.00)	24.20 (20.20–29.40)	<.001
RVSWI (mm Hg × ml × m^2^)	6.55 (4.67–9.12)	5.90 (4.40–8.00)	7.12 (5.05–9.60)	0.045

LVEDP, Left ventricular end-diastolic pressure; MPA, Main pulmonary artery; PADP, Pulmonary arterial diastolic pressure; PAMP, Pulmonary arterial mean pressure; PASP, Pulmonary arterial systolic pressure; PVR, Pulmonary vascular resistance; RAP, Right atrial pressure; RVSP, Right ventricular systolic pressure; RVSWI, Right ventricular stroke work index; SVR, Systemic vascular resistance; TPG, Transpulmonary gradient; TSG, Trans-systemic gradient.

Cardiac output (CO) and cardiac index (CI) were significantly reduced in Cluster 2 than Cluster 1, reflecting diminished global perfusion capacity. Pulmonary vascular resistance (PVR) was notably elevated (4.1 (2.56–6.4) vs. 1.4 (0.9–2.33) Wood units, *p* < 0.001), while systemic vascular resistance (SVR) was also modestly higher (24.2 (20.2–29.4) vs. 21.4 (17.6–29.4) Wood units, *p* < 0.001). Additionally, stroke volume (SV) and stroke volume index (SVI) were significantly lower in Cluster 2, consistent with advanced circulatory compromise.

Collectively, these findings underscore a more severe biventricular phenotype in Cluster 2, characterized by pronounced systolic dysfunction, elevated filling pressures, secondary valvular disease, and significant pulmonary hypertension.

### Laboratory parameters and biomarkers

Patients in Cluster 2 exhibited a laboratory profile consistent with advanced disease severity, multiorgan involvement, and worse nutritional and metabolic status (Table 4). Serum urea levels were higher in Cluster 2, yet serum creatinine levels similar in both groups. Hepatic congestion and dysfunction were more prominent in Cluster 2, with significantly elevated total (1.08 (0.72–1.58) vs. 0.58 (0.41–0.81) mg/dl, *p* < 0.001) and direct bilirubin levels (0.51 (0.31–0.84) vs. 0.21 (0.15–0.30) mg/dl, *p* < 0.001), as well as higher GGT (58.5 (30.6–103) vs. 28 (18–44) U/L, *p* < 0.001) and ALP (100 (71–131) vs. 87 (71.5–105) U/L, *p* = 0.001).

**Table 4:** Blood parameters of patients.

Variable	Overall (*n* = 524)	Cluster 1 (*n* = 282)	Cluster 2 (*n* = 242)	*p*
Urea (mg/dl)	43.0 (34.4–56.0)	40.4 (32.9–52.1)	46.4 (37.8–60.9)	<.001
Creatinine (mg/dl)	1.00 (0.83–1.20)	0.99 (0.82–1.16)	1.02 (0.84–1.22)	0.304
AST (U/L)	20.6 (15.6–27.2)	19.4 (15.4–16.4)	21.8 (16–27.7)	0.053
ALT (U/L)	20.6 (14.2–30.9)	21 (14.9–30.7)	19.9 (13.6–30.9)	0.618
Total bilirubin (mg/dl)	0.75 (0.50–1.20)	0.58 (0.41–0.81)	1.08 (0.72–1.58)	<.001
Direct bilirubin (mg/dl)	0.30 (0.19–0.54)	0.21 (0.15–0.30)	0.51 (0.31–0.84)	<.001
ALP (U/L)	90.5 (71.2–117.0)	87 (71.5–105)	100 (71–131)	0.001
GGT (U/L)	37.0 (21.0–72.0)	28 (18–44)	58.5 (30.6–103)	<.001
ProBNP (ng/L)	2232 (1000–4411)	1330 (562–2241)	3969 (2441–6595)	<.001
Total cholesterol (mg/dl)	161.2 (132.6–198.5)	184 (153–213)	140 (109–169)	<.001
Triglyceride (mg/dl)	122.0 (88.0–172.3)	147 (108–221)	96.8 (74.2–132)	<.001
HDL (mg/dl)	38.8 (32.0–48.3)	42 (36.2–50.5)	34.8 (28.1–43.3)	<.001
LDL (mg/dl)	92.3 (65.3–121.8)	107 (78–131)	80.8 (58.6–107)	<.001
Sodium (mmol/L)	138.0 (3.04)	138 (2.68)	138 (3.38)	0.015
Potasium (mmol/L)	4.5 (0.52)	4.59 (0.48)	4.39 (0.53)	<.001
Total protein (g/L)	70.8 (7.30)	72 (6.35)	69.4 (8.07)	<.001
Albumin (g/L)	43.1 (5.38)	44.9 (4.32)	41.2 (5.79)	<.001
LDH (U/L)	216.0 (185.0–265.0)	203 (177–237)	229 (202–285)	<.001
GFR (ml/min/1.73 m^2^)	81.9 (22.3)	83.5 (21.8)	80.1 (22.8)	0.080
TSH (mIU/L)	1.9 (1.2–3.1)	1.78 (1.13–2.79)	2.07 (1.33–3.46)	0.008
INR	1.2 (1.1–1.4)	1.09 (1.02–1.19)	1.33 (1.20–1.57)	<.001
HGB (g/dl)	13.8 (1.98)	14.4 (1.65)	13 (2.09)	<.001
HCT (%)	42.7 (5.53)	43.9 (4.73)	41.2 (6.02)	<.001
Platelet (10^3^/µl)	249.0 (205.0–291.0)	252 (215–289)	246 (199–293)	0.254
Lactate	1.40 (1.10–1.80)	1.3 (1.1–1.8)	1.5 (1.2–2.0)	<.001

ALP, Alkaline phosphatase; ALT, Alanine aminotransferase; AST, Aspartate aminotransferase; GFR, Glomerular filtration rate; GGT, Gamma-glutamyl transferase; HCT, Hematocrit; HDL, High-density lipoprotein; HGB, Hemoglobin; INR, International normalized ratio; LDH, Lactate dehydrogenase; LDL, Low-density lipoprotein; ProBNP, Pro brain natriuretic peptide; TSH, Thyroid-stimulating hormone.

NT-proBNP levels were nearly threefold higher in Cluster 2 compared to Cluster 1 (3,969 (2,441–6,595) vs. 1,330 (562–2,241) ng/L, *p* < 0.001), indicating greater myocardial wall stress and hemodynamic overload.

Markers of nutritional status showed significant deterioration in Cluster 2. Serum albumin (41.2 ± 5.79 vs. 44.9 ± 4.32 g/L, *p* < 0.001), total protein (69.4 ± 8.07 vs. 72.0 ± 6.35 g/L, *p* < 0.001), and HDL cholesterol levels [34.8 (28.1–43.3) vs. 42 (36.2–50.5) mg/dl, *p* < 0.001] were significantly lower, suggesting poor nutritional state and reduced hepatic synthetic function.

Hematologic findings were indicative of more pronounced anemia in Cluster 2. Both hemoglobin (13.0 ± 2.09 vs. 14.4 ± 1.65 g/dl, *p* < 0.001) and hematocrit levels (41.2 ± 6.02% vs. 43.9 ± 4.73%, *p* < 0.001) were significantly lower compared to Cluster 1, suggesting impaired oxygen-carrying capacity and potential chronic disease-related anemia.

### Cardiopulmonary exercise test (CPET) performance

Cluster 2 patients demonstrated significantly reduced functional capacity across multiple CPET parameters, consistent with more advanced heart failure physiology (Table 5). Peak oxygen consumption (peak VO_2_) was markedly lower in Cluster 2 [10.7 (9–13.2) vs. 16.0 (13.3–18.7) ml/kg/min, *p* < 0.001], reflecting impaired aerobic capacity and cardiac output reserve. Similarly, the achieved metabolic equivalents (METS) were significantly reduced [3.1 (2.6–3.8) vs. 4.6 (3.8–5.4), *p* < 0.001], indicating diminished ability to perform physical activity.

**Table 5:** Cardiopulmonary exercise test parameters of patients.

Variable	Overall (*n* = 524)	Cluster 1 (*n* = 282)	Cluster 2 (*n* = 242)	*p*
Time (Min)	6.6 (4.2–9.3)	8.41 (6.46–10.1)	5 (3.05–6.54)	<.001
Load [Work (W)]	90.0 (45.0–140.0)	130 (80–170)	55 (30–95)	<.001
VE (L/min)	46.0 (38.0–54.0)	49 (41–56)	43 (36–52)	<.001
VO_2_ (ml/min)	1072 (802–1370)	1308 (1025–1590)	835 (652–1048)	<.001
Peak VO_2_ (ml/min/kg)	13.6 (10.4–16.9)	16 (13.3–18.7)	10.7 (9–13.2)	<.001
Pred (%)	29.0 (25.1–33.9)	29.4 (25.1–35.1)	28.8 (25.3–33.0)	0.227
RER	1.03 (0.08)	1.01 (0.07)	1.05 (0.08)	<.001
METs	3.9 (3.0–4.8)	4.6 (3.8–5.4)	3.1 (2.6–3.8)	<.001
VECO_2_ slope (VE/VCO_2_)	38.0 (31.5–52.3)	33.5 (29.2–39.8)	49.1 (37.6–81.0)	<.001
VO_2_/Work slope [(ml/min)/W]	3.60 (2.02–5.27)	4.04 (2.5–5.6)	2.72 (0.45–4.6)	<.001

MET, Metabolic Equivalent; RER, Respiratory Exchange Ratio; VCO_2_, Carbon Dioxide Production; VE, Ventilation; VE/VCO_2_, Carbon Dioxide Ventilatory Equivalent; VO_2_, Oxygen Consumption; VO_2_W, Oxygen Consumption/Workload Ratio; W, Watts; Pred (%), The percentage of the patient's peak VO_2_ value during the cardiopulmonary exercise test relative to the predicted value.

Ventilatory efficiency was also substantially worse in Cluster 2, as demonstrated by a significantly elevated VE/VCO_2_ slope [49.1 (37.6–81.0) vs. 33.5 (29.2–39.8), *p* < 0.001]. Moreover, lower peak exercise oxygen pulse and VO_2_/work slope values in this group (both *p* < 0.001) further support compromised cardiovascular performance and peripheral oxygen extraction.

### Outcomes and survival analysis

**Figure 4:**
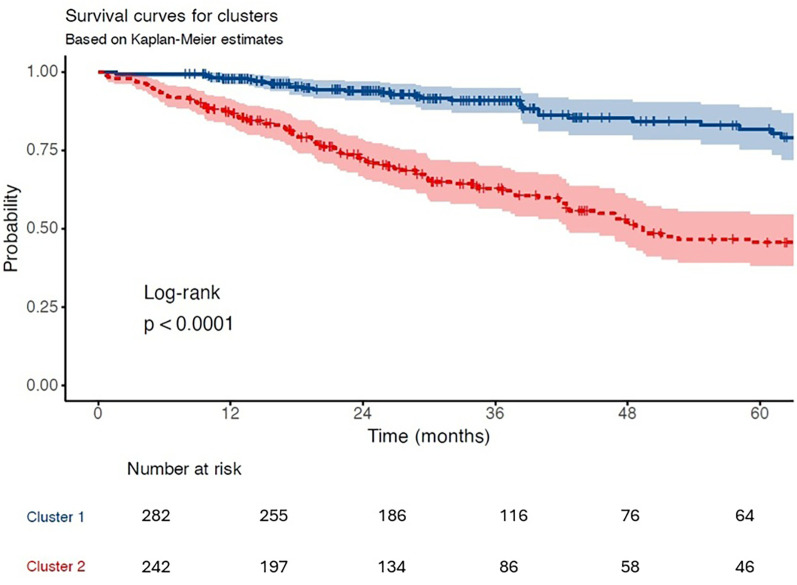
Kaplan–meier survival curves. Kaplan–Meier estimates for the composite outcome (all-cause mortality, LVAD, or transplantation) stratified by cluster assignment. Cluster 2 demonstrated significantly lower event-free survival (log-rank *p* < 0.0001).

Over a median follow-up of 2.4 years (interquartile range: 1.4–4.1), the incidence of the composite endpoint was significantly higher in Cluster 2 compared to Cluster 1 (50.0% vs. 15.6%, *p* < 0.001), highlighting the adverse prognostic profile of this subgroup (Table 6). In Cox regression analysis, assignment to Cluster 2 was associated with a 3.84-fold increased risk of experiencing the composite endpoint (hazard ratio [HR]: 3.84; 95% confidence interval [CI]: 2.72–5.43; *p* < 0.001) (Table 7, Figure 4).

**Table 6:** Clinical outcomes (LVAD implantation, heart transplantation, and death) according to phenotypic clusters.

Variable	Cluster 1 (*n* = 282)	Cluster 2 (*n* = 242)	*p*
LVAD	11 (3.9)	55 (22.7)	<.001
Tx	0 (0)	4 (1.7)	0.046
Death	34 (12.1)	79 (32.6)	<.001
Composite endpoint	44 (15.6)	121 (50.0)	<.001

LVAD, Left ventricular assist device; Tx, Heart transplantation.

**Table 7:** Cox regression analysis.

Cluster	*N* (%)	HR (CI)
1	280 (53.6)	–
2	242 (46.4)	3.84 (2.72–5.43, *p* < 0.001)

CI, Confidence Interval; HR, Hazard Ratio, *N*, Number of patients.

## Discussion

In this study, we identified two distinct phenotypic clusters—the FPC and the APC- within an advanced HF population by applying unsupervised ML to a comprehensive and multimodal dataset. These clusters exhibited significant differences in clinical profiles and were associated with long-term outcomes, underscoring their prognostic relevance.

Recent studies by Zhang et al. and Yao et al. have applied supervised ML methods to predict the need for advanced HF therapies (18, 21). Zhang et al. used data from 557 hospitalizations to develop a transparent, rule-based ML model that predicted which patients would require advanced heart failure therapies, such as LVAD or transplantation, during follow-up (18). Yao et al. introduced a novel ML framework that grouped clinical variables into flexible, overlapping categories—allowing a patient to belong partially to more than one risk group, rather than being forced into a single predefined class. This approach, inspired by fuzzy logic, better reflects the clinical continuum and supports the derivation of interpretable reasoning rules. In their pilot application, the method was tested on a real-world cohort of patients evaluated for advanced heart failure therapies, demonstrating its potential to support eligibility classification through a rule-based, interpretable design (21). While these models offer interpretable decision support, they require labeled outcomes and focus on specific treatment decisions. In contrast, our approach aimed to identify clinically meaningful phenotypes associated with prognosis, rather than just treatment eligibility. This methodology offers a broader view of clinical heterogeneity in advanced HF.

Lamp et al. used unsupervised clustering to stratify patients into five risk categories based on a composite outcome of death, LVAD implantation, transplantation, and rehospitalization over six months, and subsequently applied supervised modeling to predict these outcomes (19). Their interpretable model was trained using two distinct input sets: the invasive set, which included variables derived solely from right heart catheterization (e.g., right atrial pressure, pulmonary artery pressures, cardiac output), and the all-feature set, which combined invasive hemodynamic data with a wider range of clinical and laboratory variables. The model achieved high predictive performance, with c-statistics ranging from 0.896 to 0.969 for the invasive set, and 0.858 to 0.997 for the all-feature set, although confidence intervals were not explicitly reported. Despite the impressive discrimination, their analysis was primarily limited to hemodynamic domains. In contrast, our approach integrated a more comprehensive set of variables—including echocardiographic, cardiopulmonary exercise testing (CPET), biochemical, and invasive hemodynamic parameters—allowing for a multidimensional phenotypic characterization with prognostic relevance.

The concept of phenotyping HF patients has emerged from the need for personalized treatment. In heart failure with reduced ejection fraction (HFrEF), applying and titrating guideline-directed therapies can be challenging due to comorbidities and adverse effects on blood pressure, renal function, and electrolyte balance (22). As the HF population ages, comorbidity burden increases, reducing the feasibility of uniform treatment (“one size fit all”) approaches. Similar challenges exist in HFpEF, where pharmacological therapies have generally failed to show mortality benefit in randomized large scale trials (23–25). However, ML-based clustering studies have revealed subgroups with variable treatment responses, supporting the role of precision medicine in this heterogeneous population (26, 27).

ML has gained attraction in HF research for its capacity to manage high-dimensional data and uncover latent phenotypes not captured by traditional statistics (5, 7). Beyond providing therapeutic guidance, it also aids in risk stratification, as demonstrated by a series of analyses that applied supervised machine learning techniques to predict short- and long-term mortality with high accuracy (5, 28, 29).

**Figure 5:**
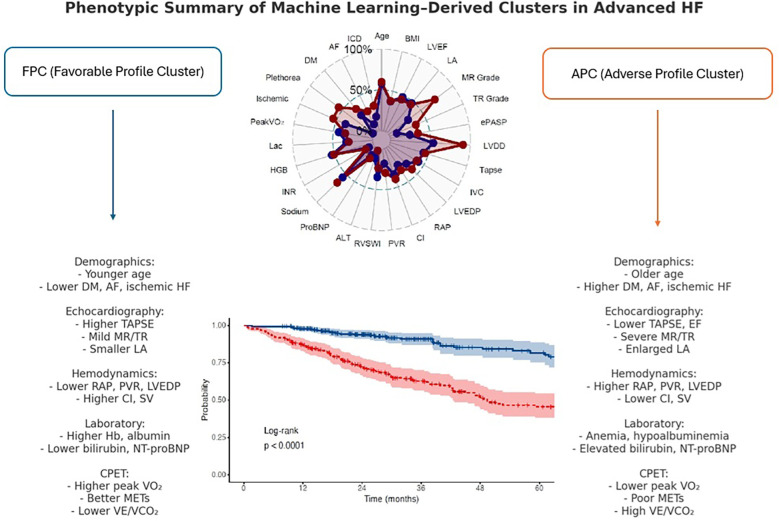
Phenotypic summary of machine learning–derived clusters in advanced HF.

Patients with advanced heart failure represent the terminal stage of the disease and often present with overlapping symptoms and complex pathophysiology (3). Although machine learning–based clustering has previously been applied in advanced heart failure, prior studies were limited by narrower variable sets, often focusing predominantly on hemodynamics or clinical data. Our study is the first to integrate a comprehensive multimodal dataset—including echocardiographic, cardiopulmonary exercise test, and invasive hemodynamic parameters—allowing a more detailed phenotypic characterization and prognostic stratification in this high-risk population. In our study, the two identified clusters—the FPC and the APC—demonstrated distinct clinical profiles (Figure 5). FPC encompassed individuals with relatively preserved hemodynamic and functional status, whereas APC represented a cohort with marked physiological deterioration and higher disease burden. These contrasting profiles were associated with markedly different long-term outcomes. Patients in the FPC group may benefit from routine follow-up and medical optimization, whereas those in the APC group may require early evaluation for advanced therapies, including inotropic support, mechanical circulatory support, or palliative care planning.

Integrating clustering outputs into clinical workflows could enable timely recognition of high-risk phenotypes and support personalized treatment strategies. As multimorbidity becomes more prevalent in HF populations, incorporating comorbidity profiles into clustering models may enhance patient stratification. Future studies should aim to externally validate these clusters and evaluate their applicability in prospective cohorts.

## Limitations

Despite the strengths of our study, several important limitations should be acknowledged. First, the sample size, although relatively large for a single-center advanced HF cohort, remains modest. Second, our cohort was predominantly male, a pattern commonly observed in advanced HF studies; nevertheless, this sex imbalance may restrict the generalizability of our findings to female patients. Third, the retrospective and observational design precludes causal inference. Fourth, although the dataset was comprehensive, it reflects a single-center experience, which may limit external validity and generalizability. While we performed repeated split-sample validation within our cohort to strengthen internal reproducibility, external validation in larger, prospective multicenter cohorts will be essential to confirm the generalizability of our findings. Fifth, binary clinical variables (e.g., sex, diabetes, atrial fibrillation, ischemic etiology) were excluded from the clustering input for methodological reasons, potentially limiting completeness of phenotyping.

## Conclusion

This study shows that unsupervised ML-based clustering can reveal clinically important phenotypes in advanced HF using routinely collected multimodal data. The identification of two distinct clusters with differing clinical profiles and outcomes highlights the potential of data-driven approaches to enhance risk stratification and guide personalized care. Prospective validation is warranted to confirm clinical utility.

## Data Availability

The raw data supporting the conclusions of this article will be made available by the authors, without undue reservation.

## Supplementary material

Supplementary Figure S1 Flowchart of the study population. Flow diagram showing patient selection. Of 653 patients evaluated at the advanced HF clinic, 129 were excluded (prior LVAD/HTx, preserved LVEF, severe pulmonary disease, contraindications to CPET/RHC, or incomplete follow-up). The final cohort included 524 patients, who were classified into two clusters (Cluster 1, n=282; Cluster 2, n=242).

Supplementary Figure S2 Missing data map. Visualization of missingness across all variables in the study cohort. Overall, 8.3% of values were missing, while 91.7% were present. The plot highlights variable- and patient-level distribution of missing data.

Supplementary Figure S3 Determination of the optimal number of clusters for hierarchical clustering. (Left) Elbow method: total within-cluster sum of squares plotted against increasing k values, with an inflection observed at k=2. (Right) Silhouette method: average silhouette width across k, peaking at k=2, supporting the choice of a two-cluster solution.

Supplementary Figure S4 PCA visualization of k-means clusters. Visualization of the two clusters identified using k-means clustering after dimensionality reduction with PCA.

Supplementary Figure S5 t-SNE visualization of k-means clusters. t-Distributed Stochastic Neighbor Embedding (t-SNE) plot depicting patient clustering based on high-dimensional input data. Clear distinction observed between Cluster 1 and Cluster 2.

Supplementary Figure S6 Cluster stability assessed by Jaccard similarity indices. Resampling-based stability analysis for the primary k-means model using continuous variables demonstrated excellent reproducibility of both clusters (Jaccard indices: 0.998 and 0.985). Sensitivity analysis with Gower distance and PAM clustering showed consistent cluster structures with moderately lower stability (Jaccard indices: 0.851 and 0.762), supporting the robustness of the identified phenotypes.

Supplementary Figure S7 Schoenfeld residuals for proportional hazards assumption. Plots of scaled Schoenfeld residuals over time for covariates included in the Cox regression models. No systematic trends were observed, indicating that the proportional hazards assumption was not violated.
