# Supplementary material for: From patterns to prognosis: machine learning–derived clusters in advanced heart failure
Source: Front Cardiovasc Med. 2025 Oct 23;12:1669538. doi: 10.3389/fcvm.2025.1669538 (PMC12589050; doi:10.3389/fcvm.2025.1669538)
Supplement: Supplementary file 1 [file Supplementaryfile1.docx]

**Supplementary Table 1. Variables Included in Clustering Analysis**

| **Variable Name** | **Type** | **Category** | **Description** |
| --- | --- | --- | --- |
| gender | Binary | Clinical/Demographic | Sex (male/female) |
| Age | Continuous | Clinical/Demographic | Age at baseline (years) |
| Height | Continuous | Clinical/Demographic | Height (cm) |
| weight | Continuous | Clinical/Demographic | Weight (kg) |
| bmi | Continuous | Clinical/Demographic | Body mass index (kg/m²) |
| ischemic | Binary | Clinical | Ischemic etiology (yes/no) |
| ht | Binary | Clinical | Hypertension (yes/no) |
| dm | Binary | Clinical | Diabetes mellitus (yes/no) |
| af | Binary | Clinical | Atrial fibrillation (yes/no) |
| hl | Binary | Clinical | Hyperlipidemia (yes/no) |
| ckd | Binary | Clinical | Chronic kidney disease (yes/no) |
| cvd | Binary | Clinical | Cerebrovascular disease (yes/no) |
| pad | Binary | Clinical | Peripheral artery disease (yes/no) |
| smoker | Binary | Clinical | Current smoker (yes/no) |
| copd | Binary | Clinical | Chronic obstructive pulmonary disease (yes/no) |
| pci | Binary | Clinical | Percutaneous coronary intervention (yes/no) |
| cabg | Binary | Clinical | Coronary artery bypass graft (yes/no) |
| icd | Binary | Clinical | Implantable cardioverter defibrillator (yes/no) |
| crt | Binary | Clinical | Cardiac resynchronization therapy (yes/no) |
| lvef | Continuous | Echocardiography | Left ventricular ejection fraction (%) |
| lvedd | Continuous | Echocardiography | Left ventricular end-diastolic diameter (mm) |
| lvesd | Continuous | Echocardiography | Left ventricular end-systolic diameter (mm) |
| la | Continuous | Echocardiography | Left atrial AP diameter (cm) |
| mrgrade | Ordinal | Echocardiography | Mitral regurgitation grade (0–3) |
| avr | Ordinal | Echocardiography | Aortic valve replacement |
| mvr | Ordinal | Echocardiography | Mitral valve replacement |
| trgrade | Ordinal | Echocardiography | Tricuspid regurgitation grade (0–3) |
| echo_pasp | Continuous | Echocardiography | Pulmonary artery systolic pressure (mmHg) |
| lvdd | Ordinal | Echocardiography | LV diastolic dysfunction grade (1-3) |
| tapse | Continuous | Echocardiography | Tricuspid annular plane systolic excursion (cm) |
| ivc | Continuous | Echocardiography | Inferior vena cava diameter (cm) |
| plethora | Binary | Echocardiography | Plethora (yes/no) |
| aortsys | Continuous | Echocardiography | Aortic systolic pressure (mmHg) |
| aortdia | Continuous | Echocardiography | Aortic diastolic pressure (mmHg) |
| aortmean | Continuous | Echocardiography | Aortic mean pressure (mmHg) |
| lvedp | Continuous | Hemodynamics | Left ventricular end-diastolic pressure (mmHg) |
| cath_pasp | Continuous | Hemodynamics | Pulmonary artery systolic pressure (mmHg) |
| cath_padp | Continuous | Hemodynamics | Pulmonary artery diastolic pressure (mmHg) |
| cath_pamp | Continuous | Hemodynamics | Mean pulmonary artery pressure (mmHg) |
| rvsp | Continuous | Hemodynamics | Right ventricular systolic pressure (mmHg) |
| rap | Continuous | Hemodynamics | Right atrial pressure (mmHg) |
| tpg | Continuous | Hemodynamics | Transpulmonary gradient (mmHg) |
| tsg | Continuous | Hemodynamics | Transsystemic gradient (mmHg) |
| sv | Continuous | Hemodynamics | Stroke volume (mL) |
| svi | Continuous | Hemodynamics | Stroke volume index (mL/m²) |
| aosat | Continuous | Hemodynamics | Aortic oxygen saturation (%) |
| mpasat | Continuous | Hemodynamics | Mean pulmonary artery oxygen saturation (%) |
| co | Continuous | Hemodynamics | Cardiac output (L/min) |
| ci | Continuous | Hemodynamics | Cardiac index (L/min/m²) |
| pvr | Continuous | Hemodynamics | Pulmonary vascular resistance (Wood units) |
| svr | Continuous | Hemodynamics | Systemic vascular resistance (Wood units) |
| rvswi | Continuous | Hemodynamics | Right ventricular stroke work index |
| glu | Continuous | Laboratory | Glucose (mg/dL) |
| urea | Continuous | Laboratory | Blood urea nitrogen (mg/dL) |
| cre | Continuous | Laboratory | Serum creatinine (mg/dL) |
| ast | Continuous | Laboratory | Aspartate aminotransferase (U/L) |
| alt | Continuous | Laboratory | Alanine aminotransferase (U/L) |
| tbil | Continuous | Laboratory | Total bilirubin (mg/dL) |
| dbil | Continuous | Laboratory | Direct bilirubin (mg/dL) |
| alp | Continuous | Laboratory | Alkaline phosphatase (U/L) |
| ggt | Continuous | Laboratory | Gamma-glutamyl transferase (U/L) |
| probnp | Continuous | Laboratory | NT-proBNP (ng/L) |
| tkol | Continuous | Laboratory | Total cholesterol (mg/dL) |
| trig | Continuous | Laboratory | Triglycerides (mg/dL) |
| ldl | Continuous | Laboratory | LDL cholesterol (mg/dL) |
| hdl | Continuous | Laboratory | HDL cholesterol (mg/dL) |
| nonhdl | Continuous | Laboratory | Non-HDL cholesterol (mg/dL) |
| sodium | Continuous | Laboratory | Sodium (mmol/L) |
| potasium | Continuous | Laboratory | Potassium (mmol/L) |
| calcium | Continuous | Laboratory | Calcium (mmol/L) |
| tprot | Continuous | Laboratory | Total protein (g/L) |
| alb | Continuous | Laboratory | Albumin (g/L) |
| ldh | Continuous | Laboratory | Lactate dehydrogenase (U/L) |
| gfr | Continuous | Laboratory | Estimated glomerular filtration rate (mL/min/1.73 m²) |
| tsh | Continuous | Laboratory | Thyroid stimulating hormone (µIU/mL) |
| pt | Continuous | Laboratory | Prothrombin time (seconds) |
| inr | Continuous | Laboratory | International normalized ratio |
| wbc | Continuous | Laboratory | White blood cell count (10³/µL) |
| hgb | Continuous | Laboratory | Hemoglobin (g/dL) |
| hct | Continuous | Laboratory | Hematocrit (%) |
| mcv | Continuous | Laboratory | Mean corpuscular volume (fL) |
| mchc | Continuous | Laboratory | Mean corpuscular hemoglobin concentration (g/dL) |
| plt | Continuous | Laboratory | Platelet count (10³/µL) |
| mpv | Continuous | Laboratory | Mean platelet volume (fL) |
| neu | Continuous | Laboratory | Neutrophils (%) |
| lym | Continuous | Laboratory | Lymphocytes (%) |
| mono | Continuous | Laboratory | Monocytes (%) |
| eos | Continuous | Laboratory | Eosinophils (%) |
| ph | Continuous | Laboratory | Blood pH |
| fo2hb | Continuous | Laboratory | Fractional O₂Hb (%) |
| fcohb | Continuous | Laboratory | Fractional COHb (%) |
| hco3 | Continuous | Laboratory | Bicarbonate (mmol/L) |
| chlor | Continuous | Laboratory | Chloride (mmol/L) |
| lac | Continuous | Laboratory | Lactate (mmol/L) |
| sure | Continuous | CPET | Exercise duration (sec) |
| load | Continuous | CPET | Exercise load (W) |
| ve | Continuous | CPET | Minute ventilation (L/min) |
| vo2 | Continuous | CPET | Oxygen consumption (mL/min) |
| peakvo2 | Continuous | CPET | Peak VO₂ (mL/kg/min) |
| predpercent | Continuous | CPET | The percentage of the patient's peak VO_2_ value during the cardiopulmonary exercise test relative to the predicted value. |
| rer | Continuous | CPET | Respiratory exchange ratio |
| mets | Continuous | CPET | Metabolic equivalents |
| hr | Continuous | CPET | Heart rate (bpm) |
| hrr | Continuous | CPET | Heart rate reserve (bpm) |
| peaksat | Continuous | CPET | Peak oxygen saturation (%) |
| veco2 | Continuous | CPET | Ventilatory efficiency (VE/VCO₂ slope) |
| vo2ws | Continuous | CPET | VO₂/work slope (mL/min/W) |
| hro2ws | Continuous | CPET | Heart rate/VO₂/work slope |

All variables were selected based on their known prognostic significance in advanced heart failure and prior phenomapping studies. Continuous variables were standardized, ordinal variables were integer-coded to preserve rank, and binary variables were excluded from K-means clustering to maintain validity of Euclidean distance calculations. To avoid multicollinearity, pairs of variables with Pearson correlation > 0.7 were identified, and one variable from each pair was removed based on clinical judgment to retain the most informative feature.

**Supplementary Table 2: Internal validation metrics (Davies–Bouldin and Calinski–Harabasz indices) across different cluster solutions.**

| **k** | **Davies–Bouldin** | **Calinski–Harabasz** |
| --- | --- | --- |
| 2 | 0.579 | 1870.16 |
| 3 | 0.605 | 1737.71 |
| 4 | 0.637 | 1657.18 |
| 5 | 0.665 | 1099.44 |
| 6 | 0.625 | 1107.82 |

**Supplementary Table 3: Split-Sample Validation Results**

Results of repeated split-sample validation (100 random replications, 70% training / 30% validation). The adjusted Rand index demonstrated good reproducibility, and prognostic separation was consistent across all replications. Pooled hazard ratio estimates further supported the robustness of the clustering solution.

| Metric | Result |
| --- | --- |
| Number of replications | 100 |
| Adjusted Rand Index (mean ± SD) | 0.77 ± 0.06 |
| Log-rank p<0.05 (across replications) | 100/100 (100%) |
| Pooled Hazard Ratio (95% CI) | 0.83 (0.78–0.89) |
| Bootstrap Jaccard stability index* | 0.99 (continuous-only variables) |

*Bootstrap Jaccard stability index already reported in the Supplementary Figure 6 (internal validation).
