# Supplementary figures and images for: From patterns to prognosis: machine learning–derived clusters in advanced heart failure
Source: Front Cardiovasc Med. 2025 Oct 23;12:1669538. doi: 10.3389/fcvm.2025.1669538 (PMC12589050; doi:10.3389/fcvm.2025.1669538)

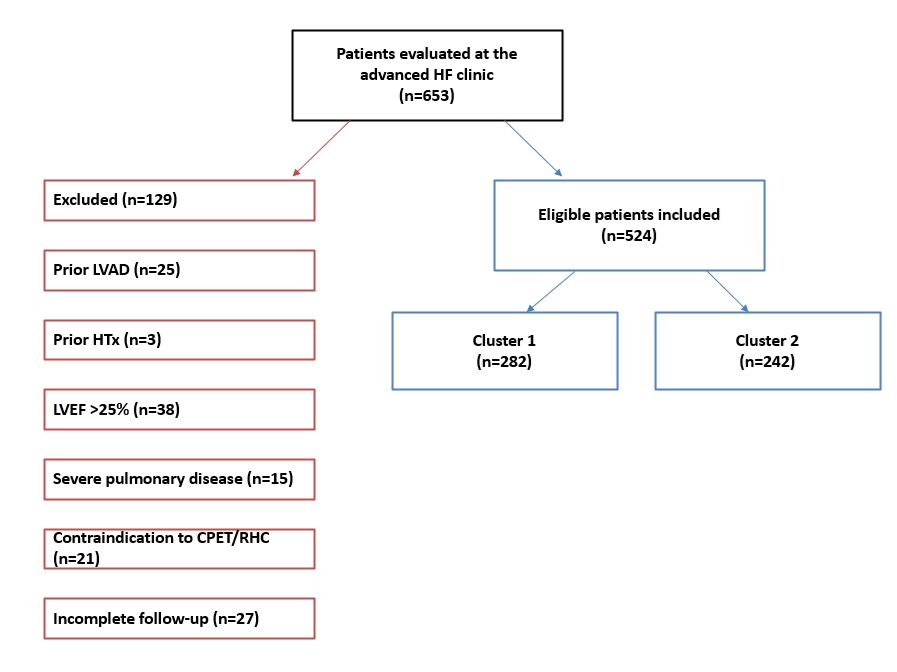

Supplement: Supplementary Figure S1 — Flowchart of the study population. Flow diagram showing patient selection. Of 653 patients evaluated at the advanced HF clinic, 129 were excluded (prior LVAD/HTx, preserved LVEF, severe pulmonary disease, contraindications to CPET/RHC, or incomplete follow-up). The final cohort included 524 patients, who were classified into two clusters (Cluster 1, n=282; Cluster 2, n=242). [file Image1.jpeg]

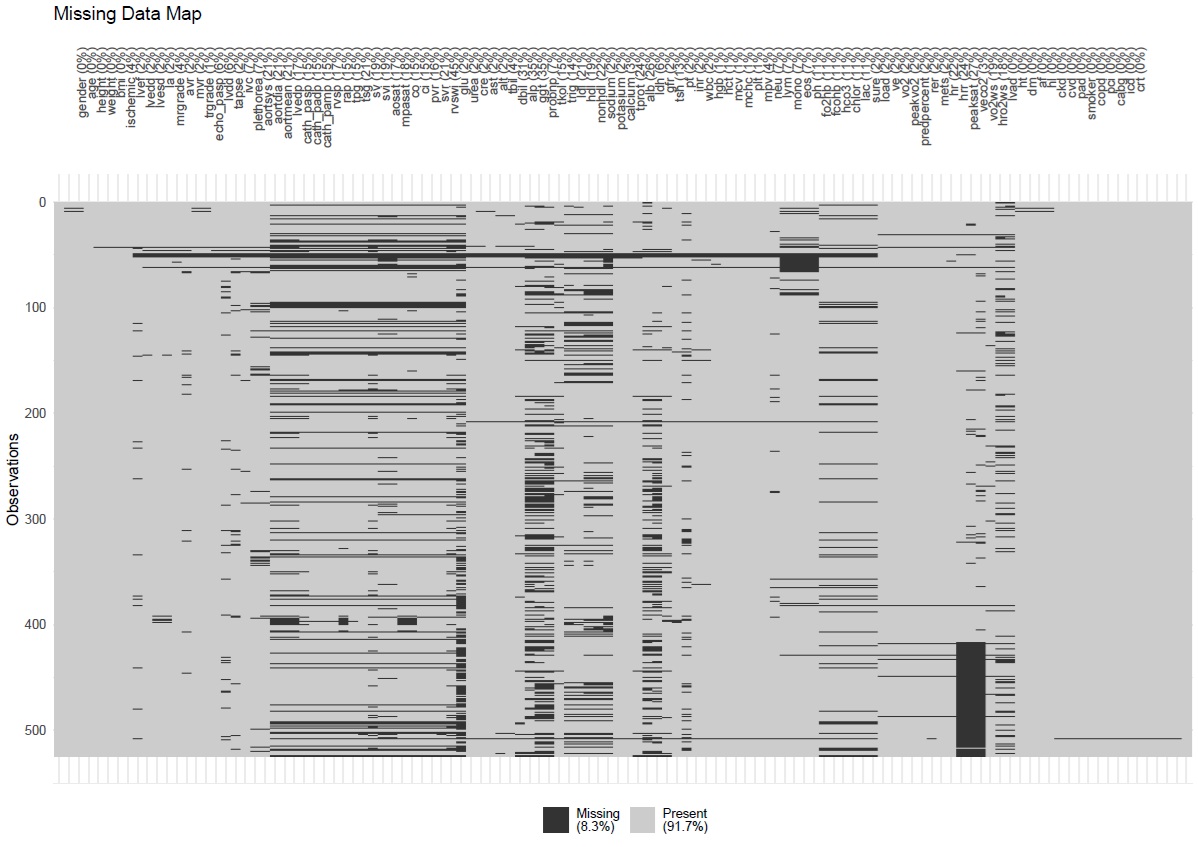

Supplement: Supplementary Figure S2 — Missing data map. Visualization of missingness across all variables in the study cohort. Overall, 8.3% of values were missing, while 91.7% were present. The plot highlights variable- and patient-level distribution of missing data. [file Image2.jpeg]

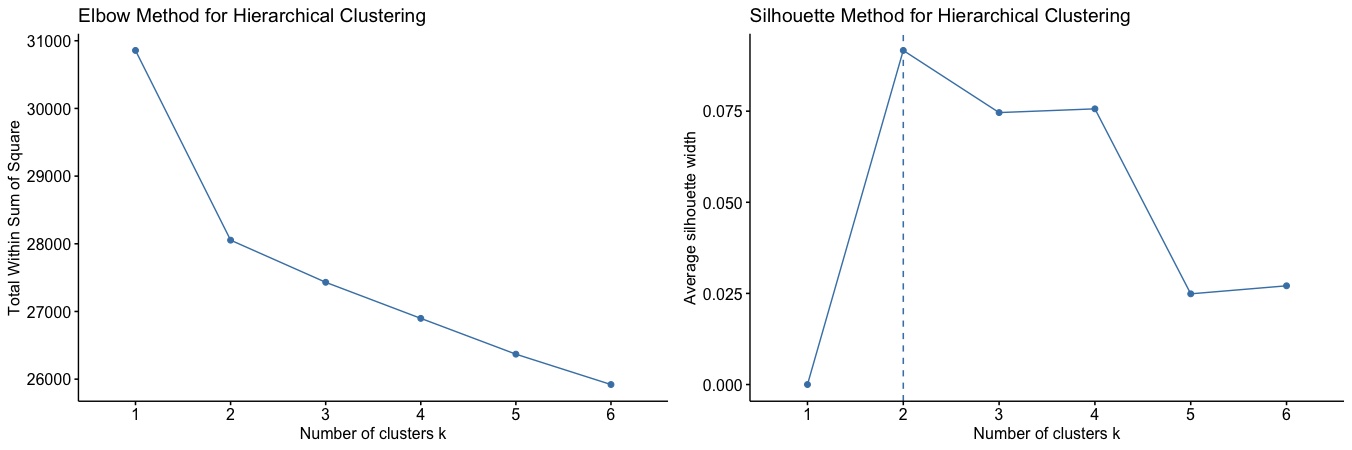

Supplement: Supplementary Figure S3 — Determination of the optimal number of clusters for hierarchical clustering. (Left) Elbow method: total within-cluster sum of squares plotted against increasing k values, with an inflection observed at k=2. (Right) Silhouette method: average silhouette width across k, peaking at k=2, supporting the choice of a two-cluster solution. [file Image3.jpeg]

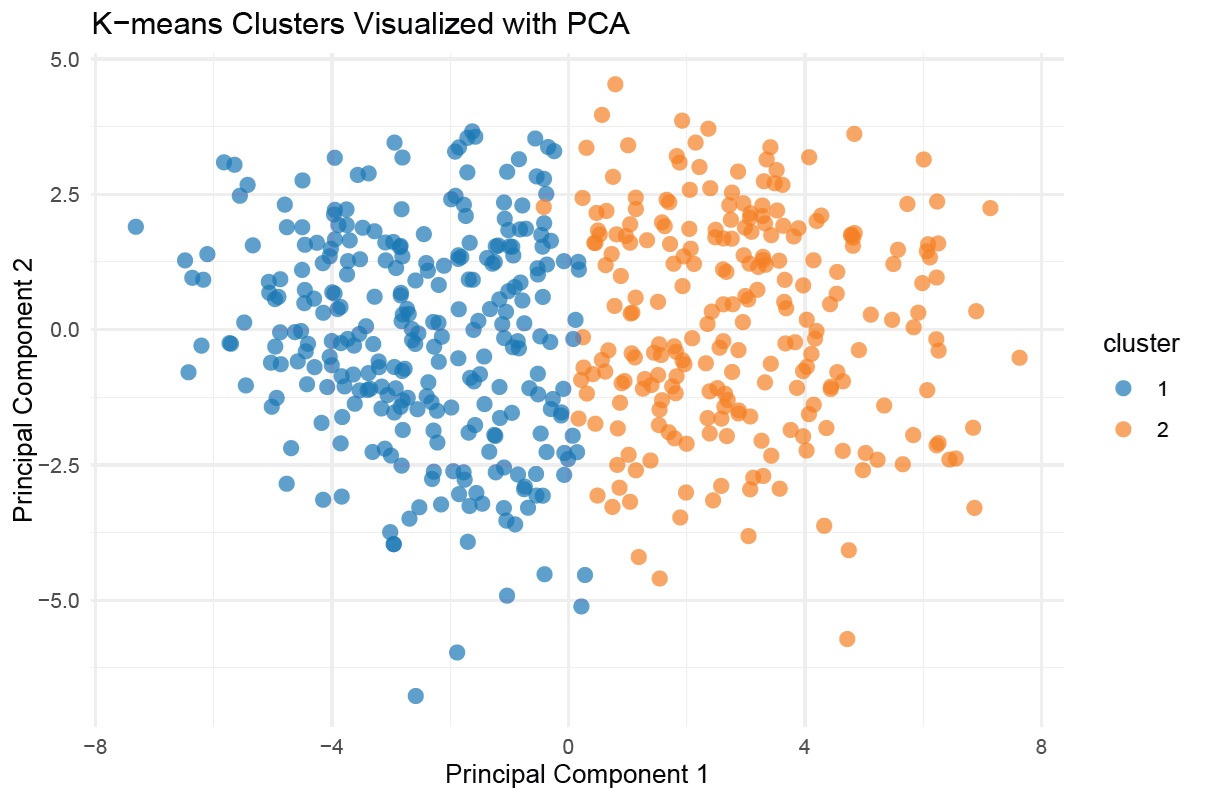

Supplement: Supplementary Figure S4 — PCA visualization of k-means clusters. Visualization of the two clusters identified using k-means clustering after dimensionality reduction with PCA. [file Image4.jpeg]

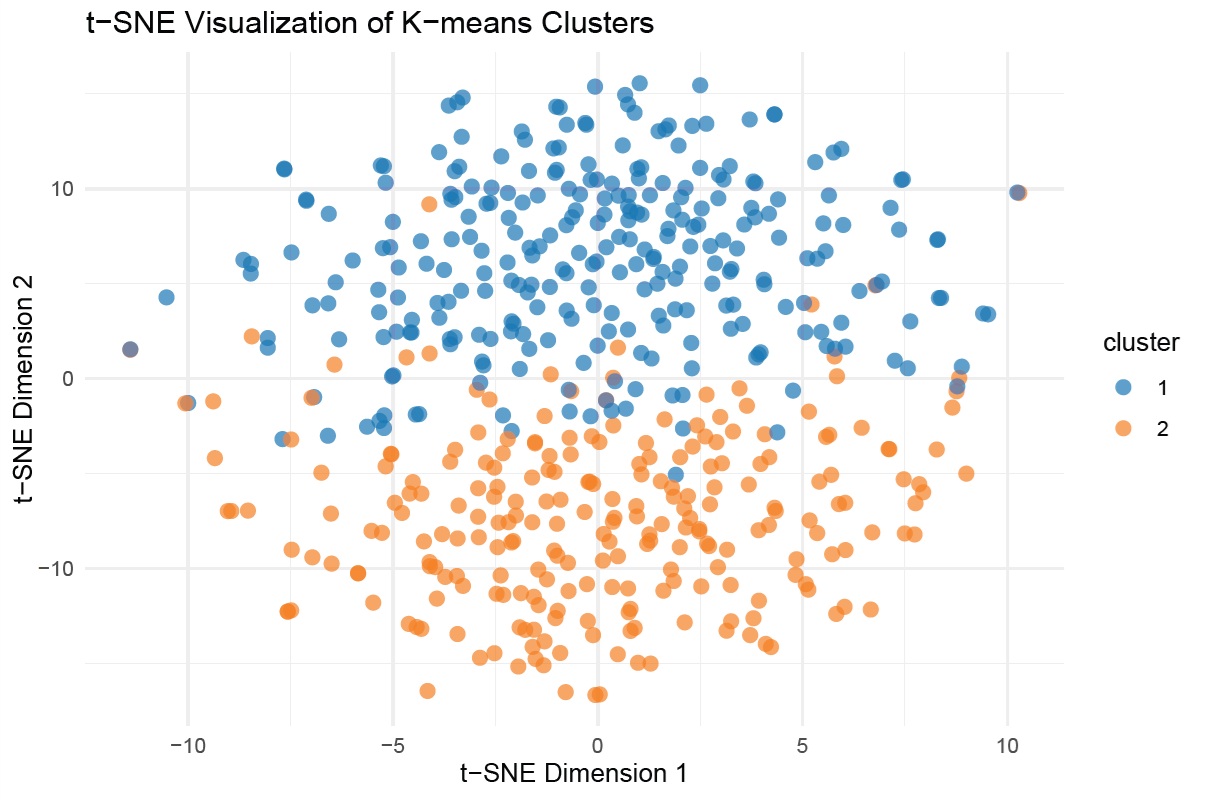

Supplement: Supplementary Figure S5 — t-SNE visualization of k-means clusters. t-Distributed Stochastic Neighbor Embedding (t-SNE) plot depicting patient clustering based on high-dimensional input data. Clear distinction observed between Cluster 1 and Cluster 2. [file Image5.jpeg]

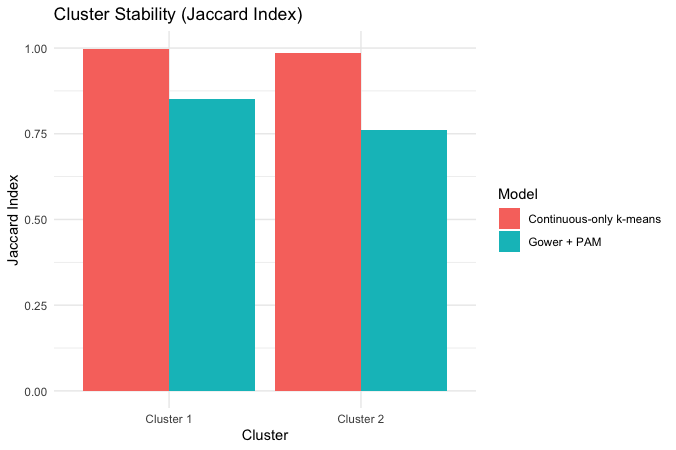

Supplement: Supplementary Figure S6 — Cluster stability assessed by Jaccard similarity indices. Resampling-based stability analysis for the primary k-means model using continuous variables demonstrated excellent reproducibility of both clusters (Jaccard indices: 0.998 and 0.985). Sensitivity analysis with Gower distance and PAM clustering showed consistent cluster structures with moderately lower stability (Jaccard indices: 0.851 and 0.762), supporting the robustness of the identified phenotypes. [file Image6.tiff]

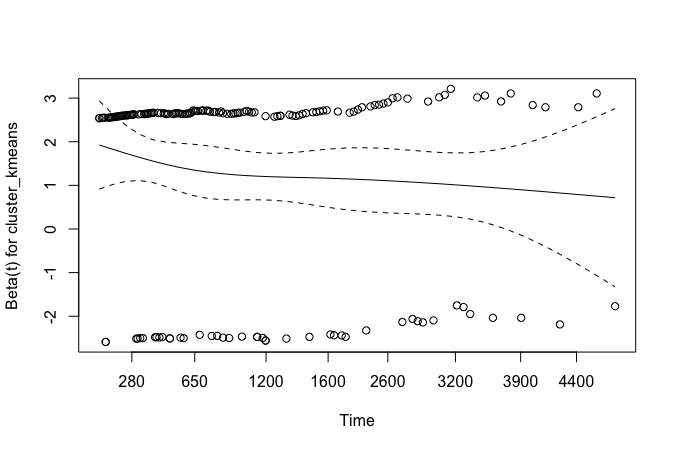

Supplement: Supplementary Figure S7 — Schoenfeld residuals for proportional hazards assumption. Plots of scaled Schoenfeld residuals over time for covariates included in the Cox regression models. No systematic trends were observed, indicating that the proportional hazards assumption was not violated. [file Image7.tiff]
